# Cultural adaptation of the mental health first aid guidelines for Brazilians with problem drinking: a Delphi expert consensus study

**DOI:** 10.1186/s12888-022-03709-5

**Published:** 2022-03-07

**Authors:** Ibrahim Ali Ayoub, Carlos Henrique Mesquita Peres, Amanda Vidotto Cerqueira, Thais Alves Assumpção, Alexandre Andrade Loch, Nicola J. Reavley

**Affiliations:** 1 Laboratorio de Neurociencias (LIM 27), Instituto de Psiquiatria, Hospital das Clinicas HCFMUSP, Faculdade de Medicina, Universidade de Sao Paulo, Sao Paulo, SP Brazil; 2 Instituto Nacional de Biomarcadores em Neuropsiquiatria (INBION), Conselho Nacional de Desenvolvimento Cientifico e Tecnológico, Sao Paulo, Brazil; 3 Centre for Mental Health, Melbourne School of Population and Global Health, University of Melbourne, Melbourne, Victoria 3010 Australia

**Keywords:** Stigma, Alcohol misuse, Delphi, Mental health first aid, Brazil

## Abstract

**Background:**

Harmful use of alcohol is highly prevalent around the world and results in a large disease burden. Most people who meet the criteria for an alcohol use disorder do not receive treatment. Those in a person’s social network can be useful in recognizing a problem and encouraging the person to seek treatment. However, many people lack the knowledge and skills to do this effectively. This study reports on the cultural adaptation for Brazil of the 2009 English-language mental health first aid guidelines for helping someone with problem drinking.

**Methods:**

A Delphi expert consensus study with two expert panels, one comprising health professionals with experience in the treatment of problem drinking and the other comprising people with lived experience was conducted. Participants rated the importance of actions to be taken to help a person with problem drinking.

**Results:**

Over two rounds, 60 participants (30 professionals and 30 people with lived experience) rated 197 items. A total of 166 items were included in the final guidelines.

**Conclusions:**

While there were many similarities with the English-language guidelines for high-income countries, the guidelines also incorporate actions of importance for Brazil, including compulsory treatment and different approaches to dealing with people with problem drinking. Further research is necessary to assess their impact.

**Supplementary Information:**

The online version contains supplementary material available at 10.1186/s12888-022-03709-5.

## Background

Consumption of alcohol is a widespread phenomenon. In 2016, an estimated 55% of the world’s population over 15 years old had consumed alcohol at some point in their lives, and 43% were current drinkers [1]. The global prevalence of heavy episodic drinking (HED) was 18.2%, with 39.5% of current drinkers engaging in HED. Also in 2016, harmful use of alcohol resulted in 5.3% of all deaths globally (which is higher than the mortality caused by diseases such as tuberculosis, HIV/AIDS and diabetes) [1], as well as 4.3% of all DALYs worldwide [2]. Among Brazilian drinkers, the prevalence of HED was 48.1%, and the prevalence of HED in the total population was 19.4%, which is higher than the global average [1]. In 2015, approximately 1.5% of the Brazilian population could be considered to have alcohol dependency [3], while in 2011 the lifetime prevalence of alcohol abuse was 10.6% and lifetime dependence was 3.6% [4].

Despite the high prevalence of drinking problems, most alcohol-dependent people do not seek professional help or participate in self-help groups, highlighting the need to develop alternative strategies to improve treatment rates [5]. Social networks may be useful in this process, as social pressure has been shown to be an important factor leading alcohol-dependent people to seek treatment [6–8], with studies suggesting that family members and friends have greater impact than trained professionals in increasing help seeking [9, 10]. However, in many cases, non-professionals may not recognize signs, may not know how to help or may offer ineffective help that differs greatly from expert recommendations [11].

In response to low levels of knowledge among the general community about how to assist someone developing a mental health problem or in a mental health crisis, the Mental Health First Aid (MHFA) course was developed to teach these skills [12]. Originating in Australia in 2000, the course has now spread to over 25 countries. A systematic review and meta-analysis of 18 controlled trials showed that MHFA training increased not only the amount of help provided to people with mental health problems, but also the knowledge of first aiders about effective treatments [13]. The MHFA course is based on expert consensus guidelines developed using the Delphi method, a technique that consists of submitting a series of statements to the evaluation and rating of experts to achieve an expert consensus [14–16]. It has been widely used in mental health research [17].

While MHFA training has been widely disseminated, this has mostly been in high-income Western countries with well-resourced health systems [18]. The appropriateness of MHFA course content and implementation models in countries with major health system and cultural differences is unknown. In general, evidence in low- and middle-income countries (LMICs) on how best to translate, adapt and scale-up population mental health interventions that have shown benefit in high-income countries is limited. A first step in this process is the development of guidelines for specific LMIC settings involving health professionals and people with lived experience from these settings.

Several Delphi studies to develop mental health first aid guidelines for middle-income countries have been conducted [19–23]. Comparison of these guidelines with those from high-income countries showed that, while there was some broad agreement across experts from different countries, there were also some culturally specific actions [24]. In Brazil, for example, alcoholism is more commonly blamed on the person and seen as a “weakness of character” than in some high-income countries [25]. Furthermore, among Brazilian healthcare professionals, people with alcohol dependency in Brazil are seen as responsible for their own condition more often than those suffering from other mental disorders [26]. Therefore, the aim of this study is to adapt the mental health first aid guidelines for helping someone with problem drinking [16] to the Brazilian cultural and health system context, in order to improve the quality of helping actions taken by someone assisting a person with problem drinking.

## Methods

This Delphi study involved four stages: (1) Questionnaire development for the Round 1 survey; (2) Panel identification and recruitment; (3) Data collection over 2 survey rounds; and (4) Guidelines development.

### Development of the questionnaire

The first step involved translating and adapting the Mental Health First Aid statements on how to help someone with problem drinking into Brazilian Portuguese. This was performed by a senior psychiatrist (AAL) and three medical students (CHMP, TAA, ACV). The translated version was checked by AAL and sent to 10 individuals who were asked to check for inconsistencies and readability of the questionnaire. There were a total of 182 statements. These were entered into an online survey website (SurveyMonkey) and grouped into categories to be displayed in sequence. Participants were asked to rate each item on a 5-point Likert scale (‘essential’, ‘important’, ‘depends/don’t know’, ‘not important’ and ‘should not be included’). At the end of each session, open-ended text boxes were included to allow the participants to add additional comments if they wished.

### Recruitment of participants

We recruited two expert panels, one comprising mental health professionals with expertise in the field of alcohol use disorders, and another comprising lay people (aged 18 years and over) with lived experience of problem drinking, either as a caregiver or as a patient with an alcohol-related disorder. For mental health professionals with experience in alcohol-related disorders, members of several university centers and hospitals were approached personally, by e-mail, or by telephone, including those of the Institute of Psychiatry of the University of Sao Paulo. For the lay participants, members of the community and of specialized help groups were approached (e.g., members of Alcoholics Anonymous). Brief information about the study was presented to them with a hyperlink to the survey, in which further instructions and explanation were included. They were also asked to send a link on to those who might also be interested. Participants were asked to tick a box indicating consent to participate before starting of the questionnaire. The study was approved by the University of Melbourne and University of Sao Paulo ethics committees.

### Data collection and analysis

In this study, the Delphi method relies on the agreement of two panels of experts rating the importance of helping statements [17]. We conducted two rounds of the survey through the aforementioned SurveyMonkey website. Participants could complete the survey in multiple sittings and in any location they desired. After the first round, statements were immediately included in the guidelines if they were endorsed by ≥80% of members in both panels as either essential or important. Statements were re-rated in the following round if they were rated as essential or important by 70–79% of either panel. Statements were immediately excluded from the guidelines if they were rated as essential or important by less than 70% of either panel.

The second round also included relevant items suggested in the open-ended comments made by the participants. After the second round, statements that received at least 80% ‘essential’ and ‘important’ ratings from both panels were included, while the remaining statements were rejected (See Additional file 1). The endorsed items then constituted the final guidelines for the Brazilian mental health first aid guidelines for helping a person with problem drinking and were written into prose (See Additional file 2).

## Results

A total of 60 participants were recruited for the first round of the study—30 professionals and 30 people with lived experience. All participants were born and lived in Brazil. The characteristics of the sample from both the first and second rounds are shown in Table 1. Overall, 50% of participants in round 1 (*n* = 60) completed round 2 (*n* = 30); the retention rate was the same for both panels.

**Table 1:** Sample characteristics

	First round (*n* = 60)	Second round (*n* = 30)
Sex		
Female, n (%)	42 (71.6%)	20 (66.7%)
Male, n (%)	17 (28.4%)	10 (33.3%)
Age, mean (SD, range)	34.85 (11.18, 18–59)	32.87 (9.09, 22–59)
Profession (professional panel)		
Nurses, n (%)	7 (23.3%)	2 (13.4%)
Doctors, n (%)	5 (16.7%)	1 (6.7%).
Psychologists, n (%)	4 (13.3%)	4 (26.7%)
Occupational therapists, n (%)	3 (10%)	3 (20%)
Workplace (professional panel), n (%)		
Hospital or healthcare center, n (%)	20 (66.7%)	6 (40%)
CAPS^a^, n (%)	6 (20%)	4 (26.7%)
Support group, n (%)	2 (6.7%)	2 (13.3%)
CVV^b^, n (%)	1 (3.4%)	1 (6.7%)
Source of experience (lay panel)		
Familial experience	9 (30%)	5 (16.7%)
Worked at hospital or clinic as non-health professional	10 (33%)	6 (20%)

^a^The CAPS’s (*Centro de Atenção Psicossocial* – Psychosocial Care Centers) are governmental treatment centers responsible for providing free mental healthcare for adults and children, including treatment for drug and alcohol use disorders [27]

^b^The CVV *Centro de Valorização da Vida*) is a non-profit organization that provides free and anonymous emotional support to people at risk of suicide [28]

**Fig. 1:**
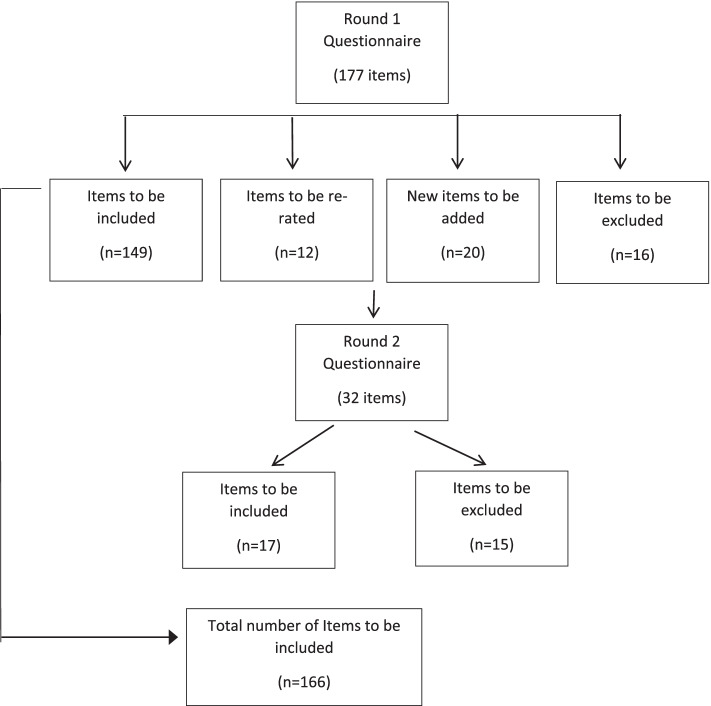
Flowchart of endorsed and rejected statements

Over the two rounds, 197 items were rated (original items plus suggestions), of which 166 were endorsed and 31 rejected (see Fig. 1).

### Differences between Brazilian and English-language guidelines

Overall, 156 items were endorsed in both the English-language and Brazilian versions of the guidelines. An additional 10 items suggested by the participants were included in the Brazilian version of the guidelines.

- Talking to the person about their drinking
  ○ The first aider should be aware that, when asked about their drinking, the person may lie about it or deny they have a problem.
  ○ The first aider should try to find out if there is a history of problem drinking in the person’s family.
  ○ If the person is experiencing withdrawal symptoms, the first aider should explain that these are part of how the body initially reacts to alcohol withdrawal.
  ○ The first aider should know the personal boundaries of the person.
  ○ The first aider should ask the person how they can help.
- Discussing professional help with the person
  ○ The first aider should explain to the person that it may be possible to take medication to reduce anxiety and to have psychotherapy to help with abstinence.
- Practical tips for low-risk drinking
  ○ The first aider should suggest that the person avoid places where alcoholic beverages or people who drink are present.
- Encouraging other supports
  ○ The first aider should promote sports and outdoor activities.
- What to do if the intoxicated person becomes aggressive
  ○ The first aider should create some distance between the person and others who may worsen the situation by taking them to an appropriate place until the situation calms down.
- Getting the intoxicated person home
  ○ The first aider should ask for professional help in case the person is unable to drive or return home because putting them in a taxi exposes them to serious risks, particularly if the person is a woman.

### Differences between panels

Eight items were approved by the professional panel but rejected by the lived experience panel with a difference of more than 10%:

- Talking to the person about their drinking
  ○ The first aider should ask the person about their drinking behaviour, e.g. about how much alcohol the person tends to drink.
  ○ The first aider should not accuse the person of being an alcoholic.
- What to do if the person unwilling to get professional help
  ○ If the person is unwilling to get professional help because they don’t want to stop drinking completely, the first aider should explain that the treatment goal may be to reduce alcohol consumption rather than to quit altogether.
- Practical tips for low-risk drinking
  ○ The first aider should ask the person if they would like some tips on low-risk drinking.
  ○ If the person wants some advice on low-risk drinking, the first aider should advise the person what a standard drink is.
  ○ If the person wants some advice on low-risk drinking, the first aider should inform the person that the number of standard drinks is often listed on the beverage’s packaging.
  ○ If the person wants some advice on low-risk drinking, the first aider should advise the person to reduce the amount of alcohol they drink by consuming drinks with lower alcohol content (for example, drinking light beer instead of full strength beer).
  ○ If the person wants some advice on low-risk drinking, the first aider should advise the person to switch to non-alcoholic drinks when they start to feel the effects of alcohol.

Nine items were approved by the lived experience panel but rejected by the professional panel with a difference of more than 10%:

- Talking to the person about their drinking
  ○ The first aider should talk to the person when both are in a calm frame of mind.
  ○ The first aider should ask the person how the person feels the day after heavy drinking.
  ○ The first aider should show the person that consuming excess alcohol is not only causing the person itself harm, but also to those around them.
- General principles for emergencies related to alcohol intoxication
  ○ If the intoxicated person stops breathing, the first aider should be aware they need expired air resuscitation (EAR).
- What to do if the person unwilling to get professional help
  ○ The first aider should try to convince the person to accept treatment even when the person does not believe they need an intervention.
  ○ The first aider should know that alcohol may undermine the person’s reasoning faculties and should consider compulsory treatment as an option.
  ○ The first aider should proceed with compulsory treatment if the person is a risk to themselves or others.
- Encouraging other supports
  ○ The first aider should suggest self-help groups to the person.
- What to do if the intoxicated person becomes aggressive
  ○ The first aider should verify if the person is aggressive due to abstinence.

## Discussion

The aim of the study was to culturally adapt the mental health first aid guidelines for helping a person with problem drinking used in English-speaking countries. To our knowledge, this is the first study conducted in Brazil using a systematic process of cultural adaptation to improve supportive behaviours towards people with problem drinking.

Many similarities between the Brazilian guidelines and the English-language guidelines were seen. The endorsement rate of initial statements included in the Round 1 questionnaire was high (84.2%, 149 out of 177 statements being endorsed), suggesting a high level of agreement. In the second phase, another 7 items from the English-language guidelines were endorsed resulting in 156 original items being endorsed (84.7%).

While both the professional and lay panels opted to endorse statements in agreement with the guidelines of the English-speaking countries relating to taking a calmer, less forceful approach, including avoiding arguing with an intoxicated person, the lay panel rejected the statement pertaining to not labelling the person an ‘alcoholic’ or ‘addict’. This could suggest that although many Brazilians still see people with problem drinking as somehow guilty for their own condition [29], those that have personal experience with problem drinking or work as mental healthcare professionals have different perceptions. However, it should be noted that stigma towards individuals with mental disorders has not been as extensively studied in Brazil as in English-speaking (or other high-income) countries [30], and any conclusion might be premature.

In the second round, many statements about low-risk drinking were rejected, mainly due to low levels of endorsement by the lived experience panel, several of whom came from Alcoholics Anonymous. This suggests that non-professional Brazilians that deal with harmful alcohol use or dependence are more reluctant to use a risk reduction strategy than those from English-speaking countries, while professionals consider it a valid approach. One participant from the lived experience panel stated: “There is no such thing as a use pattern that does not offer risks, therefore, I do not agree with low-risk drinking”. On the contrary, a participant from the professional panel suggested even more risk reduction strategy statements. In the first round, the statement *If the person is unwilling to get professional help, because they don’t want to stop drinking completely, the first aider should explain that the treatment goal may be to reduce alcohol consumption rather than to quit altogether* was also rejected by the lived experience panel, corroborating the results of the second round. This shows that professional training and experience lead to the belief that alcohol can be consumed in a safe manner even by those with a history of problem drinking [31], a counter-intuitive thought that may not be disseminated among lay people. An advantage of the Delphi method for developing guidelines is that they consist of items that both those who favor risk reduction and those who favor abstinence can agree are essential or important.

A number of statements about compulsory treatment were endorsed by the lived experience panel but rejected by the professional panel, such as *The first aider should proceed with compulsory treatment if the person offers risk to herself or others* and *The first aider should try to convince the person to accept treatment even when the person does not believe they need an intervention*. This implies that non-professionals are more likely than professionals to see compulsory treatment as a viable option, and to suggest treatment even when the person appears to be unwilling to undergo it, a situation that may reflect the controversies in involuntary treatment in psychiatry [32]. In Brazil, involuntary treatment has been the center of many discussions in the “Brazilian Psychiatric Reform” policy [33], possibly reducing its acceptance among professionals. Such items do not appear in the English version of the guidelines, showing greater acceptance of involuntary treatment among Brazilians, especially those who are not professionally trained.

### Strengths and limitations

The major limitation of this study relates to the smaller number of participants when compared to other Mental Health First Aid Delphi studies [14, 15] due to the low (50%) retention rate. A key strength relates to the inclusion of two different panels, one for professionals and another for people with lived experience, allowing for a greater range of views on alcohol use and development of actions that were acceptable not only to professionals, but also to those who have first-hand experience with problem drinking. Studies that incorporate the views of people with lived experience are less common in Brazil than in high-income countries and may play a role in building capacity in advocacy movements.

## Conclusions

Through the use of the Delphi expert consensus method involving Brazilian mental health professionals and people with lived experience, a culturally adapted set of guidelines for how to provide mental health first aid to someone with problem drinking were produced. While there were many similarities with the English-language guidelines for high-income countries, the guidelines also incorporate actions of importance for Brazil, including the discussion about compulsory treatment, the promotion of alternative activities (e.g. sports and outdoors activities) to prevent drinking and a different approach for talking to the person about their drinking.

The guidelines may be disseminated as a stand-alone product or used as the basis for MHFA training in Brazil, thus helping to improve knowledge and helping behaviours of the public towards people with problem drinking. This may contribute to early intervention and better outcomes for people with problem drinking. Further research is necessary to evaluate this.

## Supplementary Information

**Additional file 1.** Statements that were presented to the panels and their ratings across 3 rounds of the survey.

**Additional file 2.** Expert consensus guidelines in Portuguese.

## Data Availability

The data supporting our findings is attached as the Additional file, which contains all the statements that were presented to the panels and their endorsement rates.

## Abbreviations

MHFA: Mental Health First Aid
LMICs: Low- and middle-income countries
