# Supplementary material for: Cultural adaptation of the mental health first aid guidelines for Brazilians with problem drinking: a Delphi expert consensus study
Source: BMC Psychiatry. 2022 Mar 7;22:168. doi: 10.1186/s12888-022-03709-5 (PMC8900433; doi:10.1186/s12888-022-03709-5)
Supplement: Supplementary file 2 — Additional file 2. Expert consensus guidelines in Portuguese. [file 12888_2022_3709_MOESM2_ESM.docx]

**O que é problema com bebida?**

Problema com bebida refere-se ao consumo de álcool em níveis suficientes para causar danos a curto ou longo prazo. Esses danos incluem:

- Problemas familiares ou sociais (ex: relacionamento, trabalho, problemas financeiros)
- Ferimentos não letais e letais (ex: como resultado de acidentes, quedas, violência, acidentes automobilísticos)
- Problemas de saúde mental (ex: ansiedade, depressão)
- Problemas de saúde física (ex: desde náusea e dores de cabeça até úlceras gástricas e doença no fígado ou coração)

A pessoa pode ter motivos para beber que devem ser abordados. Por exemplo, ela pode estar bebendo para lidar com problemas pessoais, como dificuldades no relacionamento ou problemas mentais não tratados. É importante entender o histórico familiar de consumo de álcool da pessoa, pois isto pode interferir na abordagem. Problemas mentais podem ser causados ou aumentados pelo álcool. Por favor, veja outros guidelines nessa série para ter mais informações sobre como ajudar alguém com outros tipos de problemas mentais.

**Tipos de problema com bebida:**

1. **Beber pesado episódico (ou *binge drinking*):** refere-se a beber em uma ocasião pelo menos 5 doses-padrão de álcool para homens ou 4 doses-padrão para mulheres. A dose-padrão não foi padronizada no Brasil, variando de 10g a 14g de álcool.
2. **Intoxicação por álcool**: são as alterações comportamentais e psicológicas geradas pelo consumo recente de álcool, envolvendo:
   - Comportamento agressivo ou inapropriado
   - Humor instável
   - Julgamento prejudicado
   - Fala arrastada, incoordenação
   - Possível coma
3. **Transtorno por uso do álcool:** envolve consumo de álcool problemático por pelo menos 12 meses, em que ocorrem pelo menos dois dos seguintes critérios:
   - Consome álcool em quantidade maior que o pretendido
   - Tenta repetidamente parar de beber, mas falha
   - Gasta muito tempo procurando álcool, utilizando álcool ou se recuperando dos efeitos do álcool
   - Tem fissura ou desejo forte pelo álcool
   - Não consegue desempenhar funções no trabalho ou escola por causa do álcool
   - Continua consumindo álcool mesmo que isso gere problemas sociais ou pessoais
   - Reduz atividades sociais, profissionais ou de lazer para beber
   - Consome álcool em situações que isso pode gerar risco de vida
   - Continua consumindo álcool mesmo percebendo que isso é um problema ou que isso aumenta um problema físico ou psicológico
   - Tem tolerância, que é a redução no efeito do álcool e a necessidade de usar doses cada vez maiores
   - Tem abstinência quando para de utilizar o álcool, ou consome álcool ou remédios para aliviar a abstinência

Adaptado de: Manual diagnóstico e estatístico de transtornos mentais (DSM-V)

**Falando com alguém sobre seu consumo de álcool**

Se você estiver preocupado com o consumo de álcool de alguém, fale com ela de forma aberta e honesta. Fale com ela em um ambiente quieto e privado em um momento em que não haverá interrupções. Fale com ela de uma maneira apoiadora ao invés de ameaçar, confrontar ou dar sermões a ela. Porém, tenha em mente seus próprios limites ao abordá-la.

Tente entender a percepção da própria pessoa sobre seu consumo de álcool. Pergunte a ela sobre o quanto ela bebe (porém tenha em mente que ela pode mentir a respeito), e se ela acredita que seu consumo de álcool é um problema. Tente ouvir a pessoa sem julgamentos e evite expressar julgamentos morais sobre seu consumo de álcool.

Considere a prontidão da pessoa para falar sobre seu consumo de álcool, perguntando sobre as áreas de sua vida que podem estar sendo afetadas, como seu humor, desempenho no trabalho e relacionamentos. Esteja ciente de que a pessoa pode não reconhecer ou admitir que tem um problema com álcool, e que tentar força-la a admitir isso pode causar conflitos.

Identifique e converse sobre o comportamento da pessoa ao invés de criticar o seu caráter - por exemplo: dizer "Seu hábito de beber parece estar interferindo com as suas amizades" ao invés de "Você é um bêbado patético".

Quando você estiver falando sobre o consumo de álcool de uma pessoa, tenha em mente que ela pode se recordar de uma forma diferente de eventos que aconteceram enquanto estava sob efeito do álcool. Ela pode inclusive não se lembrar de certos eventos.

Pergunte à pessoa como ela acredita que você possa ajudar, e diga para ela o que você está disposto a fazer para ajudar. Isso pode variar desde ser um bom ouvinte até organizar ajuda profissional. Não espere que a pessoa mude rapidamente sua forma de pensar ou de se comportar; essa conversa pode ser a primeira vez que a pessoa pensou sobre seu hábito de beber como um problema.

**Encorajando a pessoa a mudar seu padrão de consumo**

Embora somente a própria pessoa possa fazer a decisão para mudar seu consumo, é possível que você a ajude. Mudar o hábito de beber não é fácil. Você deve saber que:

- A força de vontade e determinação de uma pessoa nem sempre são suficientes para parar seu uso problemático de álcool.
- O aconselhamento de forma isolada pode não ajudar a pessoa a mudar seus hábitos de uso de álcool.
- A pessoa pode tentar parar de beber ou mudar seu comportamento mais de uma vez antes de obter sucesso
- Muitas mudanças no estilo de vida são necessárias para mudar o padrão de consumo

Diga à pessoa que somente ela mesma é responsável pela redução do seu consumo de álcool e que, embora mudar o padrão de consumo seja difícil, ela deve continuar tentando. Se parar de beber não for o objetivo da pessoa, reduzir a quantidade de álcool consumido também pode ser um objetivo válido.

Caso a pessoa deseje dicas de como reduzir o consumo de álcool, ofereça algumas dicas da caixa *“Dicas práticas para reduzir o consumo de álcool”*.

**Dicas práticas para reduzir o consumo de álcool**

- Oriente onde ela pode conseguir informações sobre reduzir o consumo de álcool e como acessar tais informações
- Aconselhe à pessoa que não permita que outros encham seu copo antes de ela terminar a bebida, para que ela não perca a conta de quanto álcool consumiu
- Aconselhe a pessoa a beber bastante água numa ocasião em que estiver bebendo álcool para não desidratar.
- Aconselhe a pessoa a tomar uma bebida de cada vez.
- Aconselhe a pessoa a evitar competições e jogos de bebidas
- Aconselhe a pessoa a gastar o tempo livre em atividades que não envolvam beber.
- Aconselhe a pessoa a evitar acompanhar seus amigos a cada bebida
- Aconselhe a pessoa a ter noção do teor alcoólico de sua bebida
- Aconselhe a pessoa a identificar as situações em que está mais sujeita a beber e evitar tais situações se possível
- Aconselhe a pessoa a evitar locais com muita bebida ou pessoas que a forcem a beber

**Reduzindo os riscos associados com a bebida**

Para reduzir os riscos associados com a continuação do hábito de beber, encoraje a pessoa a encontrar informações de como reduzir os danos causados pelo consumo de álcool. Também, se apropriado, aconselhe a pessoa de que o álcool pode interagir com drogas ilícitas ou remédios de forma inesperada, o que pode levar a uma emergência médica. Se a pessoa estiver grávida ou amamentando, aconselhe ela de que parar completamente a com a bebida é a melhor forma de não prejudicar o bebê.

**Encorajando outros suportes**

Encoraje a pessoa a contactar amigos e familiares que apoiam seus esforços de mudar seu consumo de álcool. Conscientize-a de que nem todos os amigos e familiares apoiarão seus esforços, e sugira que passe tempo com aqueles que não bebem. Além disso, conscientize-a sobre a variedade de grupos de apoio para problemas com bebida (ex: grupos de auto-ajuda). Incentive atividades físicas, esportes e atividades ao ar livre.

**Lidando com a pressão social para beber**

É comum que haja pressão social para ficar bêbado quando consumindo álcool. Aconselhe a pessoa a ser assertiva quando ela se sentir pressionada a beber mais do que deseja ou pretende. Diga para a pessoa que ela tem o direito de recusar álcool. Sugira diferentes formas da pessoa dizer não, tais como dizer “não, obrigado” sem dar mais explicações, “eu não quero”, “eu não me sinto bem” ou “estou tomando remédio”. Estimule a pessoa a praticar essas diversas formas de dizer não. Sugira para a pessoa que as pessoas que se importam com ela irão aceitar sua decisão de não beber ou beber menos.

**E se a pessoa não estiver disposta a mudar seu consumo de álcool?**

Se a pessoa não quiser mudar seu hábito de bebida, você pode falar com um profissional da saúde para determinar como abordar a pessoa sobre suas preocupações, ou você pode consultar outros que já lidaram com problemas com álcool sobre formas eficazes de ajudar. Outra estratégia é conversar com a pessoa sobre a relação entre seu consumo de álcool e as consequências negativas que ela vem sofrendo. Se a pessoa não estiver disposta a mudar seu hábito de bebida, não beba junto a ela.

**Ajuda profissional**

| **Quando procurar ajuda profissional?** |
| --- |
| A pessoa pode precisar de ajuda profissional com o seu hábito de beber quando ela:   - Reconhece que pensa muito sobre álcool e sobre quando terá sua próxima chance de beber - Está endividada por causa da quantidade de dinheiro que gasta com álcool - Torna-se ansiosa quando não consegue ter acesso a álcool - Precisa de álcool para ajudá-la a lidar com determinadas situações - Envolve-se em brigas/discussões ou acidentes por causa do álcool - Tem dificuldade em executar as tarefas do dia a dia   **O que fazer se a pessoa não estiver disposta a procurar ajuda profissional?**  Quando ajuda profissional é sugerida pela primeira vez, a pessoa pode ter dificuldade em aceitar que precisa de ajuda, ou pode não querer ajuda. Assegure a pessoa que a ajuda profissional é confidencial.  Se a pessoa continuar resistente em procurar ajuda profissional, continue sugerindo isso se ela estiver colocando a si mesma ou outros em risco.  Esteja preparado para falar com a pessoa sobre procurar ajuda profissional no futuro. Seja compassivo e paciente enquanto espera que ela aceite que precisa de ajuda. Procurar ajuda profissional é em última instância decisão da pessoa. Ela não pode ser forçada, exceto em certas situações, por exemplo, após uma emergência médica ou após um episódio de violência que resulta em um chamado da polícia.  **Primeiros-socorros para intoxicação por álcool ou abstinência**  **Intoxicação por álcool e abstinência**  *Intoxicação por álcool* refere-se ao nível significativamente elevado de álcool no sangue que prejudica o pensamento e comportamento da pessoa. Enquanto intoxicada, a pessoa pode se envolver em diversas atividades de risco (como sexo desprotegido, brigas ou dirigir embriagado).  Sinais da intoxicação por álcool incluem:   - Perda de coordenação - Fala arrastada - Desequilíbrio e quedas - Comportamento agressivo ou beligerante - Vômitos - Sonolência   A intoxicação por álcool pode atingir níveis perigosos e até levar à morte. A quantidade de álcool necessária para isso difere de pessoa para pessoa.  *Abstinência de álcool* refere-se aos diversos sintomas que podem ocorrer a uma pessoa que estava bebendo pesada e regularmente para de beber ou começa a beber menos. Não é uma simples ressaca. É importante explicar para a pessoa sobre abstinência e sobre a possibilidade de que um médico receite remédios para auxiliar no processo, bem como psicoterapia.  **O que fazer se a pessoa estiver intoxicada?** |
| *Fique calmo e se comunique de forma adequada*  Fale com a pessoa de forma respeitosa, usando linguagem clara e simples. Não ria, zombe ou provoque a pessoa. Não tente conversar seriamente com uma pessoa sobre seu hábito de beber enquanto ela estiver intoxicada. Se a pessoa ficar agressiva, existem algumas coisas adicionais que você deve considerar (veja o quadro: *“O que fazer se a pessoa ficar agressiva”*).  Afaste pessoas que estiverem piorando a situação ou leve a pessoa para um local reservado.  *Fique atento ao perigo*  Avalie a situação para danos potenciais e certifique-se de que a pessoa, você mesmo e os outros estão seguros. Saiba que a pessoa pode estar mais intoxicada do que ela imagina. Enquanto a pessoa estiver intoxicada, ela está em maior risco de ser agredida física ou sexualmente. Observe a pessoa e seus entornos para prevenir quedas. Pergunte para a pessoa se ela tomou algum remédio ou outras drogas, para o caso de sua condição virar uma emergência médica. Também saiba que o consumo de álcool pode mascarar a dor de ferimentos. Observe a pessoa para sinais de aumento de agressividade.  *Mantenha a pessoa segura*  Fique com a pessoa ou certifique-se de que ela não seja deixada sozinha. Mantenha ela longe de máquinas ou objetos perigosos. Se a pessoa tentar dirigir um veículo ou andar de bicicleta, desencoraje ela (por exemplo, falando para ela sobre os riscos para si e para os outros). Somente impeça a pessoa de dirigir se for seguro para você fazê-lo. Caso contrário, chame a polícia.  Se a pessoa expressar pensamentos suicidas ou demonstrar comportamento suicida, chame um serviço de emergência.  Se você achar que a pessoa é um risco para si mesma, providencie que ela vá ao hospital; caso contrário, organize um transporte seguro para leva-la para casa. Não coloque-a sozinha em um taxi pois isso a expõe a risco; se necessário, chame uma ambulância.  **Eu posso ajudar a pessoa a melhorar da intoxicação?**  Somente o tempo tornará a pessoa sóbria novamente. O corpo metaboliza aproximadamente uma dose padrão de álcool por hora. Beber café, dormir, andar ou tomar banhos frios não acelera o processo.  **Emergências médicas**  **O que fazer se a pessoa ficar agressiva**  Se a pessoa ficar agressiva, verifique o risco a você mesmo, à própria pessoa e aos outros. Garanta sua própria segurança a todo momento para que você continue podendo ajudar de forma efetiva.  Se você se sentir inseguro, procure ajuda de outras pessoas. Não fique com a pessoa intoxicada se você estiver sob perigo. Fique calmo e tente acalmar a situação com as seguintes técnicas:   - Fale de uma maneira calma e não conflituosa - Fale devagar e com confiança em um tom de voz gentil e acolhedor - Tente não provocar a pessoa; evite falar de forma hostil ou ameaçadora e evite brigar com ela - Use palavras positivas (como “fique calmo”) ao invés de palavras negativas (como “não brigue”), pois palavras negativas podem gerar uma resposta mais agressiva - Considere interromper a conversa para deixar a pessoa se acalmar um pouco - Tente fornecer à pessoa um ambiente silencioso longe de barulho e distrações   Se você estiver em um lugar fechado, tente manter as saídas abertas para que a pessoa não se sinta encurralada e você ou terceiros consigam sair facilmente se necessário.  Se ocorrer uma violência, procure auxílio emergencial.  É importante saber que intoxicação e abstinência de álcool podem levar a emergências médicas.  **Sinais de uma emergência relacionada ao álcool**   - A pessoa está vomitando continuamente - A pessoa está inconsciente (ex: dormiu e não pode ser acordada) - Você suspeita de intoxicação grave pois a pessoa apresenta um desses sintomas:   - respiração irregular, superficial ou lenta   - pulso irregular, fraco ou lento   - pele fria, pegajosa, pálida ou azulada - Você suspeita que alguma droga tenha sido adicionada à bebida (ex: a pessoa fica muito intoxicada rapidamente) - A pessoa tem um possível traumatismo craniano (ex: caiu e bateu a cabeça) - A pessoa demonstra sinais de abstinência grave, como:   - tem febre   - fica delirante ou confusa   - convulsiona   - tem alucinações   **O que fazer em uma emergência médica?** |
|  |

Em uma emergência médica, chame uma ambulância ou leve a pessoa para o hospital. Não tenha medo de procurar ajuda médica para a pessoa, mesmo que isso implique em problemas legais para ele.

Se uma ambulância for chamada ou ajuda médica for buscada, certifique-se de que a pessoa:

- Não seja deixada sozinha
- Não receba alimentos ou bebidas pois ela pode engasgar se não estiver completamente consciente
- Seja mantida aquecida para evitar hipotermia (embora a pessoa possa sentir-se quente, sua temperatura corporal pode estar diminuindo)
- Tenha suas vias aéreas, respiração e circulação monitoradas
- Seja colocada em posição lateral de segurança (PLS)

É benéfico que um amigo ou familiar acompanha a pessoa ao hospital, visto que pode fornecer informações importantes à equipe de saúde.

**Outros princípios de primeiros socorros para manter em mente**

- Se a pessoa estiver vomitando e estiver consciente, mantenha-a sentada. Alternativamente, coloque-a em posição de lateral de segurança (PLS)
- Se a pessoa estiver sem pulso, ela precisará de ressuscitação cardiopulmonar (RCP)

**Posição lateral de segurança**

Toda pessoa inconsciente precisa de auxílio médico imediato, e deve ter suas vias aéreas mantidas abertas. Se ela for deixada deitada de costas, ela pode sufocar no próprio vômito, ou sua língua pode bloquear sua respiração. Procure por vidro quebrado ou outros objetos cortantes no chão antes de virar a pessoa para a posição lateral de segurança (PLS). Colocar a pessoa na PLS ajuda a manter as vias aéreas abertas. Se necessário, limpe as vias aéreas depois que ela vomitar.
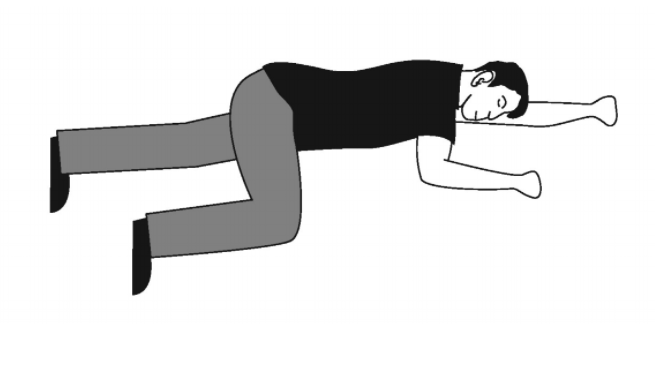


**Objetivo desse guideline**

Esse guideline foi criado para ajudar membros do público a fornecer primeiros socorros a alguém que experimentou um evento traumático. O papel do socorrista é ajudar a pessoa até que auxílio profissional chegue ou a crise se resolva.

**Desenvolvimento desse guideline**

Esse guideline é baseados nos conhecimentos de pessoas com experiência de vida em problemas com bebida (consumidores e cuidadores) e profissionais de saúde mental (médicos, pesquisadores e educadores) da Austrália, Canadá, Alemanha, Irlanda, Países Baixos, Nova Zelandia, Suécia, Suíça, Reino Unido, Estados Unidos da America e Brasil.

**Como usar esse guideline**

É importante que você ajuste sua ajuda às necessidades da pessoa que você está ajudando. Esses guidelines são apenas um conjunto de recomendações gerais.

Esse guidelines foi desenvolvido como parte de uma coleção de guidelines sobre como ajudar uma pessoa com problemas de saúde mental. Esses outros guidelines podem ser baixados em:

mhfa.com.au/resources/mental-health-first-aid-guidelines

Embora esses guidelines tenham direitos autorais, eles podem ser livremente reproduzidos para fins não comerciais, desde que a fonte seja citada. Por favor, cite esse guideline da seguinte forma:

*[Reference]*

Questionamentos devem ser enviados para: mhfa@mhfa.com.au
